# Interventions to Reduce Mental Health Stigma Among Health Care Professionals in Primary Health Care: A Systematic Review and Meta-Analysis

**DOI:** 10.3390/ijerph22091441

**Published:** 2025-09-17

**Authors:** Lazzat Zhamaliyeva, Nurgul Ablakimova, Assemgul Batyrova, Galina Veklenko, Andrej M. Grjibovski, Sandugash Kudaibergenova, Nursultan Seksenbayev

**Affiliations:** 1 Department of General Practice No. 2, West Kazakhstan Marat Ospanov Medical University, Aktobe 30012, Kazakhstan; lzhamalieva@mail.ru; 2 Department of Pharmacology, Clinical Pharmacology, West Kazakhstan Marat Ospanov Medical University, Aktobe 030012, Kazakhstan; 3 Department of Propedeutics of Internal Disease, West Kazakhstan Marat Ospanov Medical University, Aktobe 030012, Kazakhstan; gvictor63@mail.ru; 4 Reaviz Universtiy, Saint Petersburg 198095, Russia; a.grjibovski@yandex.ru; 5 Department of Epidemiology and Modern Vaccination Technologies, I.M. Sechenov First Moscow State Medical University (Sechenov University), Moscow 119048, Russia; 6 Department of Healthcare Organization and Preventive Medicine, North-Eastern Federal University, Yakutsk 677000, Russia; 7 Department of Health Policy and Management, Al-Farabi Kazakh National University, Almaty 050040, Kazakhstan; 8 Department of General and Applied Psychology, Al-Farabi Kazakh National University, Almaty 050040, Kazakhstan; sandugash.kudaibergenova@kaznu.kz; 9 Department of Psychiatry and Addiction Medicine, Semey Medical University, Semey 071400, Kazakhstan; nursultan.zhaksylykovich@smu.edu.kz

**Keywords:** mental health, depression, stigma, intervention, primary care, healthcare professionals, mental disorders, doctor, nursing

## Abstract

Background: Stigmatizing attitudes toward individuals with mental health conditions are common among healthcare professionals in primary healthcare (PHC) settings, posing a major barrier to early diagnosis, appropriate treatment, and recovery. Methods: This systematic review and meta-analysis evaluated the effectiveness of interventions aimed at reducing mental health-related stigma among PHC professionals (general practitioners, nurses, community health workers, and allied providers). Eligibility was restricted to interventional studies targeting PHC staff; non-clinical populations and students without clinical practice were excluded. Comparators included usual training, waitlist control, or pre–post evaluation. A systematic search of PubMed, Scopus, and Web of Science was conducted in accordance with PRISMA guidelines, and the protocol was registered in PROSPERO (CRD420251074412). Results: Twenty-five studies met the inclusion criteria, of which three contributed to the quantitative synthesis. Interventions included educational, contact-based, and multicomponent approaches. Risk of bias was assessed using tools appropriate to study design. Interventions generally improved knowledge and attitudes and, to a lesser extent, behavioral intentions. Meta-analysis of pre–post changes using the Opening Minds Scale for Health Care Providers (OMS-HC) demonstrated a significant reduction in stigma (MD = −0.27, 95% CI −0.40 to −0.14; *p* < 0.001; I^2^ = 91%). A difference-in-differences analysis of studies with intervention and control groups confirmed this effect with moderate heterogeneity (MD = −0.18, 95% CI −0.25 to −0.11; *p* < 0.0001; I^2^ = 50%). Conclusions: Contact-based and multicomponent interventions were associated with stronger and more sustained effects. The main limitations of the evidence were short follow-up periods, reliance on self-reported outcomes, methodological heterogeneity, and the possibility of publication bias. Our findings suggest that reducing stigma among PHC professionals can enhance patient engagement, timely diagnosis, and quality of care in routine clinical practice.

## 1. Introduction

Mental health disorders represent a significant and growing global burden. According to the 2021 Global Burden of Disease Study, there were approximately 970 million cases of mental disorders in 2019, marking a 48% increase from 1990 [1]. Anxiety and depression remain the most common, contributing to 17% of all years lived with disability in 2021 [2]. The Coronavirus Disease 2019 pandemic further exacerbated this trend: global prevalence of depression and anxiety increased by about 28%, adding some 54 million and 83 million additional cases, respectively, in 2020 [3]. This rise in mental health conditions places a substantial strain on healthcare systems and economies. Despite this, significant treatment gaps prevail: estimates show between 35% and 85% of individuals with mental conditions receive no care, especially in low- and middle-income countries; however, two thirds of this population may not seek help due to stigma and discrimination [4]. Primary healthcare (PHC) functions as the initial point of contact for most individuals seeking health services. Accordingly, the integration of mental health into PHC has been identified as a central strategy within the World Health Organization’s (WHO) Mental Health Gap Action Programme (mhGAP). However, stigma among health professionals often undermines early detection and quality management of mental illness. Stigmatizing attitudes toward individuals with mental disorders are frequently reported among healthcare professionals across both primary and secondary levels of care. In particular, such attitudes are prevalent among physicians working in primary care settings [5,6,7]. For example, a recent study comparing healthcare professionals across primary and secondary care settings revealed that physicians and nurses working in PHC exhibited more negative attitudes toward individuals with severe mental disorders than their counterparts in secondary care [8].

Stigmatization encompasses a wide range of negative attitudes and behaviors toward individuals with mental disorders and has been recognized within healthcare systems and among professionals as a major barrier to treatment and recovery [9]. It contributes to reduced empathy, patient avoidance, diagnostic delays, and inadequate referrals, ultimately lowering the quality of care, diminishing patient trust and adherence, and placing additional strain on health services [5,7].

Evidence supports several effective stigma-reduction strategies. Educational interventions, particularly those incorporating contact with persons with lived mental health experience, consistently demonstrate moderate positive effects on changing attitudes and intentions to treat. A 2021 meta-analysis identified direct and indirect contact combined with education as among the most effective approaches for healthcare professionals [10]. Moreover, recent reviews have emphasized emerging complementary methods, such as mindfulness training, digital learning, and organizational policy reforms that reinforce stigma reduction [11]. However, long-term sustainability of interventions remains uncertain, with many studies lacking extended follow-up.

Several systematic reviews and meta-reviews over the last five years lend further insight. A 2020 network meta-analysis ranked social contact plus education highest for improving provider attitudes and behavioral intentions [10]. A more recent 2024 systematic review focused on healthcare professionals highlighted the medium effectiveness of contact-based interventions, observing smaller impacts on knowledge retention and stressing a need for methodological consistency [12]. A 2022 meta-review noted variability in study quality and called for more longitudinal data to assess durability of outcomes [13].

Given the growing prevalence of mental disorders, the integration of mental health within PHC, and the well-established role of stigma in undermining care quality, there is a pressing need for focused synthesis of interventions targeting stigma among PHC providers. In this review, we aimed to address the following research question: “What is the effectiveness of interventions aimed at reducing mental health-related stigma among primary healthcare professionals, and how do these interventions vary across intervention types and settings with respect to their impact on knowledge, attitudes, and behavioral outcomes?”. In contrast to previous systematic reviews and meta-analyses that primarily focused on either physicians, nurses, or medical students, our study aims to include all categories of healthcare professionals working in PHC settings. By evaluating their implementation strategies, outcome measures, and sustainability, this review seeks to provide evidence-based recommendations for integration into routine clinical practice.

## 2. Materials and Methods

This systematic review was conducted in accordance with the Preferred Reporting Items for Systematic Reviews (PRISMA) guidelines (Supplementary Table S1). The review protocol was prospectively registered in the International Prospective Register of Systematic Reviews (PROSPERO) under registration ID CRD420251074412.

### 2.1. Search Strategy

We conducted a comprehensive search of the Web of Science, Scopus, and PubMed databases on 13 July 2025, without applying date restrictions. Only studies published in English were included. Various search strategies were developed based on the PICOS framework and are presented in Table S2. We employed Boolean operators (OR and AND) to combine terms within sets.

### 2.2. Inclusion and Exclusion Criteria

The selection of eligible studies was guided by the PICOS criteria, following the approach recommended by the Centre for Evidence-Based Medicine, University of Oxford (UK) [14]. Five search concepts were combined in order to capture relevant literature: primary care (e.g., PHC, outpatient, family medicine, ambulatory care), PHC staff (e.g., doctors, nurses, mental health professional or clinical psychologist); stigma (e.g., mental health stigma, discrimination or stereotyping, attitude); mental health (e.g., depression, anxiety or mental disorder); intervention (e.g., education, training, contact-based education). We applied filters to include only peer-reviewed articles published in English. Studies published as reviews, protocols, or conference abstracts were excluded from the analysis.

Participants: This review focused on healthcare professionals providing direct patient care in PHC settings. Eligible participants included general practitioners (GPs) (family physicians), nurses, nurse practitioners, community health workers, mental health professionals or counselors based in PHC, and allied health professionals such as psychologists and social workers practicing within PHC. Additionally, medical and nursing students engaged in clinical practice were considered. There were no restrictions regarding participants’ age, gender, professional experience, or country. To be included, studies had to report stigma-related outcomes specifically attributable to healthcare professionals, not to patients, caregivers, or the general public. Studies were excluded if they targeted non-clinical populations (e.g., administrators, caregivers), students not yet in clinical roles, or healthcare workers practicing exclusively in secondary or tertiary care settings unless data for PHC staff were reported separately.

Intervention: The interventions under investigation were those aimed at reducing stigma related to mental illness among PHC professionals. Eligible interventions included: educational interventions: Structured lectures, seminars, and knowledge-based modules on mental health; training programs: Practical or professional trainings, whether in-person or online, focused on stigma reduction skills; communication-focused interventions: Initiatives involving patient interaction training, including contact-based components or role-playing; mental healthcare education: Programs specifically addressing provider knowledge, perception, and care approaches toward mental health conditions; peer support interventions: Programs involving individuals with lived experience of mental illness or peer-based support structures among healthcare staff.

Excluded interventions were those not specifically focused on mental health stigma (e.g., general stress management), not targeted at healthcare professionals, or unrelated to mental illness (e.g., stigma associated with human immunodeficiency virus), obesity, or disabilities unless mental illness was explicitly addressed). Additionally, mass media campaigns, theoretical papers, and non-empirical reports (e.g., protocols or editorials) were excluded.

Outcome: The primary outcomes of interest were changes in healthcare professionals’ knowledge, attitudes, and behaviors related to mental health stigma. These outcomes were assessed using validated scales or qualitative indicators of change, such as reductions in discriminatory practices, increased empathy, or improvements in intention to provide care to individuals with mental illness. Secondary outcomes included qualitative findings exploring the design, implementation, or perceived effectiveness of stigma-reduction interventions. Studies were included if they reported either measurable pre–post changes or in-depth thematic analysis related to intervention outcomes. Studies that did not report outcomes specific to healthcare providers, or that measured public or patient-level stigma only, were excluded.

For the purpose of synthesis, studies were grouped a priori by intervention type (educational, contact-based, multicomponent/system-level), professional group (GPs, nurses, CHWs, allied providers), and study design (RCTs, quasi-experimental, pre–post). Outcomes were categorized into knowledge, attitudes, and behaviors.

### 2.3. Data Selection and Extraction

All records identified through database and manual searches were managed using EndNote 21 (Clarivate Analytics, Philadelphia, PA, USA) for reference management, de-duplication, and initial sorting of records. Retrieved articles were categorized into separate folders according to their source, and duplicate entries were subsequently removed. AB and NA independently screened the titles and abstracts of the identified studies. Prior to full screening, the reviewers calibrated their approach on a subset of records to ensure consistency of inclusion and exclusion decisions. In cases of disagreement, a third researcher (LZh) was consulted, or the issue was resolved through discussions involving all authors.

### 2.4. Statistical Analysis

A meta-analysis was conducted to evaluate the impact of interventions aimed at reducing mental health stigma among healthcare workers, using the OMS-HC. To standardize results across studies, all total scores were converted to average item scores, based on the 15-item structure of the scale. Two separate meta-analyses were performed: Pre–post analysis included all intervention groups that reported both pre- and post-intervention data, regardless of the presence of a control group. The effect size was calculated as the mean difference between post- and pre-intervention scores, with standard errors derived from pooled standard deviations and post-intervention sample sizes. Difference-in-differences (DiD) analysis was applied to studies that included both intervention and control groups. The effect size was calculated as the net change in the intervention group minus the net change in the control group: (post-pre in intervention)—(post-pre in control). This approach controls for external time-related factors. Random-effects models were used for both analyses. Heterogeneity was assessed using the I^2^ statistic. Funnel plots were generated to evaluate the presence of publication bias, and Egger’s test was applied when at least three studies were available (pre–post analysis only). All statistical analyses were conducted using RStudio (version 2024.12.1 Build 563), an integrated development environment developed by Posit, PBC (formerly RStudio, Inc., Boston, MA, USA). Meta-analyses were performed using the meta package (version 8.1-0) for conducting and visualizing meta-analyses. A random-effects model was chosen because included studies varied in design, intervention type, professional groups, and outcome measures, making it unlikely that they were a part of one general population and estimated a common underlying effect size. This approach allowed us to account for between-study heterogeneity and provide more conservative pooled estimates compared to a fixed-effect model. Given the small number of studies available for meta-analysis, formal sensitivity analyses (e.g., leave-one-out procedures or restriction to specific study designs) were not feasible. Instead, robustness of the findings was considered qualitatively by comparing the direction and magnitude of effects across included studies.

### 2.5. Risk of Bias Assessment

The risk of bias for each included study was assessed using validated tools appropriate for the respective study design. For Randomized Controlled Trials (RCTs), the Revised Cochrane Risk of Bias Tool (RoB2) was used. In the case of cluster randomized trials, the dedicated RoB2 for Cluster-Randomized Trials version was applied. Studies with a pre–post design without a control group were evaluated using the NIH Quality Assessment Tool for Before-After (Pre–Post) Studies with No Control Group, developed by the U.S. National Heart, Lung, and Blood Institute. For quasi-experimental studies (e.g., non-randomized studies with control groups), the Risk of Bias in Non-randomized Studies of Interventions (ROBINS-I) tool was used, following Cochrane recommendations. For mixed-methods studies, the Mixed Methods Appraisal Tool (MMAT), version 2018, was used to assess the quality of both quantitative and qualitative components as well as the integration between them. For purely qualitative studies, the Critical Appraisal Skills Programme Qualitative Checklist (CASP) was employed to evaluate methodological rigor and relevance. Each study was independently assessed by two reviewers. Discrepancies were resolved through discussion or consultation with a third reviewer.

Certainty of evidence was assessed qualitatively, considering study design, risk of bias, consistency of findings across studies, directness of evidence to the PHC context, and precision of estimates. Although we did not apply the Grading of Recommendations Assessment, Development and Evaluation (GRADE) framework formally, these domains informed the interpretation of the strength of evidence in our review.

## 3. Results

### 3.1. Study Selection

**Figure 1:**
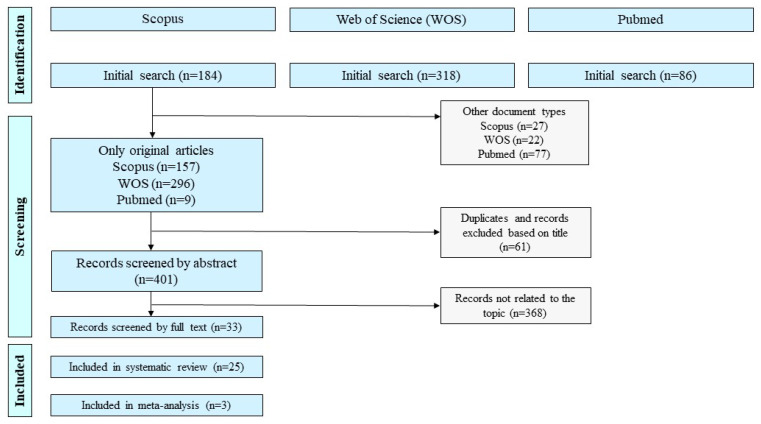
PRISMA flow chart.

A total of 588 relevant studies were retrieved from the databases (Figure 1). After removing non-original records and duplicates, 401 studies (68.2%) were screened by abstract. Following screening of 33 full-texts, 25 studies were deemed eligible for inclusion in the qualitative synthesis, with 3 of these also included in the quantitative synthesis. The most common reasons for exclusion at the full-text stage were: studies not conducted among PHC professionals, interventions not directly targeting mental health stigma, and absence of relevant outcome measures.

### 3.2. Study Characteristics

Among the included studies, a variety of research designs were employed to evaluate the effectiveness of interventions. RCTs were used in 11 studies, including 7 cluster RCTs, 2 individually RCTs, and 2 pilot RCTs. These studies typically included control groups and pre–post measurements to assess intervention efficacy. Pre–post studies without control groups were the most frequent design, found in 8 studies. Quasi-experimental designs were reported in 2 studies. Mixed-methods designs combining qualitative and quantitative approaches were used in 4 studies, often incorporating focus groups, interviews, and surveys to capture both measurable outcomes and contextual understanding (Table 1).

### 3.3. Interventions

The 25 included studies utilized a range of interventions designed to reduce mental health stigma among healthcare professionals in PHC settings. These interventions varied in complexity, mode of delivery, and underlying mechanisms. Based on their key components, they can be grouped into several thematic categories.

Training-based educational interventions, which primarily involved structured teaching sessions to improve mental health knowledge and attitudes, were the most common. These interventions often utilized lectures, workshops, and interactive discussions focused on stigma, diagnosis, treatment, and communication [15,16,17,18,19,20,21].

Multi-component or comprehensive interventions integrated educational training with supervision, Community Representatives, team-based planning, or systems-level change. These interventions often targeted structural stigma alongside individual beliefs. A significant number of these interventions were based on or adapted from the WHO mhGAP guidelines. The mhGAP framework provided a structured approach to recognizing and managing common mental disorders in low-resource primary care settings, while embedding anti-stigma messaging. Interventions based on mhGAP frequently included training modules on communication, clinical decision-making, and stigma reduction, and were often combined with supervision, peer learning, or contact with persons with lived experience [22,23,24,25,26,27,28]. Other multicomponent approaches include studies Browne et al. and Rosendal et al. [29,30].

Contact-based anti-stigma interventions involved direct or indirect interaction with people with lived experience (PWLE) of mental illness. These interventions emphasized narrative storytelling, co-facilitation, and role modeling to humanize mental illness and reduce social distance [25,31,32,33,34,35].

The use of audiovisual materials and simulation modeling, in which video recordings, thematic discussions, role-plays, or clinical simulation exercises were employed to enhance realism and emotional engagement in learning. These methods were used to foster empathy, improve clinical decision-making, and reinforce anti-stigma messages [17,21,22,25,35,36].

Interventions incorporating psychological reflection and emotional processing aimed to shift implicit biases by fostering self-awareness, empathy, and cultural sensitivity. These often involved facilitated reflection, emotional intelligence training, or trauma-informed approaches [29,31,32,37].

Interventions with longer-term or sustained engagement, including booster sessions, longitudinal supervision, or follow-up training, were designed to enhance the durability of intervention effects over time [18,24,25,26,27,30,36].

### 3.4. Outcomes of Interest

Across the included studies, a variety of standardized tools were employed to assess outcomes related to mental health stigma among healthcare professionals in primary care settings. These outcomes primarily focused on changes in knowledge, attitudes, and behaviors toward people with mental disorders, measured both immediately post-intervention and at follow-up.

Stigma-related attitudes were the most frequently assessed domain. The Opening Minds Scale for Health Care Providers (OMS-HC) was used in multiple studies to evaluate attitudinal stigma [31,32,33,37]. This 15-item scale captures key dimensions such as disclosure, help-seeking, and social distance. Several studies employed the Mental Illness: Clinicians’ Attitudes Scale (MICA) to assess implicit negative beliefs and stereotypes among clinicians [16,24].

In the Chilean context, a locally developed instrument was used to assess health professionals’ attitudes. Grandon et al. employed the Escala de Actitudes de los Profesionales de Salud hacia Personas con Trastornos Mentales scale (EAPS-TM), which is based on the MICA scale and adapted to the sociocultural and linguistic context of Chile. The EAPS-TM comprises two subscales: Stigmatizing Beliefs and Infantilization and Relational Distance, and demonstrated good internal consistency in the study (α = 0.79 and α = 0.70, respectively) [35].

In a study evaluating stigma outcomes among primary care providers (PCPs), public health professionals (PHPs), and community representatives (CRs), the Attitudes to Mental Illness Questionnaire (AMIQ) was used. Scores were collected at baseline, post-training, and at 6-month follow-up. Notably, PCPs demonstrated a moderate improvement in attitudes, increasing from a mean score of 0.59 to 1.94. Improvements were primarily observed on two items related to understanding and empathy. Internal consistency of the AMIQ for PCPs improved from α = 0.32 at baseline to α = 0.74 at endline [23]. In the study evaluating the Recovery Speaks intervention, several validated tools were used to assess stigma-related outcomes among primary care providers. These included the Affective Reaction Scale (emotions), Social Distance Scale (SDS) measuring social avoidance, Attribution Questionnaire (stereotypes, fear, coercion), Recovery Knowledge Inventory (recovery-oriented knowledge), and Competence Assessment Instrument (self-rated competence). Significant improvements were observed post-intervention in reduced negative stereotypes, fear, avoidance, and increased sense of competence [34].

The Depression Attitude Questionnaire (DAQ) and its revised version Revised-Depression Attitudes Questionnaire (R-DAQ) were used in three studies to assess changes in healthcare providers’ attitudes toward depression. The scales capture key dimensions such as professional confidence, therapeutic optimism, and beliefs about recognition and treatment of depression. In Haddad et al., significant post-training improvements were found in confidence and optimism, sustained at 9 months [36]. In Payne et al., Screen, Care, Advise, and decide on Next steps (SCAN) training led to increased confidence and more positive attitudes toward nurses’ role in treating depression and psychotherapy [18]. In Oladeji et al., R-DAQ results showed sustained gains at 6 months in confidence and recognition-related attitudes, and a notable decline in therapeutic pessimism [27]. These findings highlight the DAQ/R-DAQ as useful tools for capturing attitude shifts following depression-related training.

The SDS was used in four studies to assess reductions in stigma through changes in healthcare professionals’ willingness to interact with individuals with mental illness. In Flanagan et al. (2016), SDS scores in the intervention group decreased from 4.0 to 3.5, while remaining unchanged in the control group, suggesting a moderate improvement [34]. In Mittal et al. (2020) [32], the SDS was used to assess stigma (5 items, 4-point scale, total score 5–20; lower scores indicate less stigma). Internal consistency was high (α = 0.80–0.90). While the group-by-time interaction was not significant, the main effect of time was significant (*p* = 0.023), indicating a reduction in social distance following the intervention.

In Kaiser et al. (2022) [26], SDS was adapted for use in Nepal, showing that a greater proportion of reducing stigma among health providers (RESHAPE) trained participants demonstrated a large reduction in social distance compared to the training as usual (TAU) group [26].

In Kohrt et al. (2023) [25], a cluster RCT comparing RESHAPE and, TAU SDS scores significantly decreased in the RESHAPE group by −10.6 points (95% CI: −14.5 to −6.7), compared to −2.8 (95% CI: −8.3 to 2.7) in the control, indicating a substantial effect of the training intervention on stigma reduction [25]. These findings support the SDS as a robust measure of interpersonal stigma, sensitive to change following structured anti-stigma training programs across diverse cultural settings.

Knowledge outcomes were assessed using tailored tools aligned with the intervention content. The Mental Health Knowledge Schedule was used in several studies to evaluate general knowledge about mental health [24]. In other cases, authors developed mhGAP-aligned knowledge tests to assess familiarity with WHO-recommended protocols [17].

Rossendal et al. (2005) used a pre- and post-intervention design (1 month before and 12 months after training) to evaluate GPs attitudes. The training led to reduced negative attitudes toward patients with somatoform disorders (e.g., less anxiety, greater enjoyment in working with such patients) and increased professional confidence, though only a few items reached statistical significance after Bonferroni correction. No formal stigma scales were used; outcomes were based on Visual analogue scales (VAS) and Likert responses to custom-designed items [30].

Roussy et al. (2015) [38] reported that participants at the intervention site rated consumer-led training more positively than clinician-led training in terms of relevance (*p* < 0.05), though differences in overall training outcomes did not reach statistical significance (*p* = 0.067). No validated stigma scale was used; outcomes were based on participant perceptions of training quality and relevance.

In the study by Shirazi et al., the researchers used a customized assessment tool based on Diagnostic and Statistical Manual of Mental Disorders, Fourth Edition (DSM-IV) criteria to evaluate GPs’ performance via unannounced standardized patients [20].

In the study evaluating the Equipping PHC for Equity (EQUIP) intervention, staff self-confidence in delivering equity-oriented healthcare (EOHC) was assessed using a 6-item questionnaire. Each item was rated on a 0–10 scale, reflecting confidence in addressing trauma, equity, and discrimination in clinical care. Confidence was measured pre-intervention, after educational sessions, and post-intervention. Significant improvements were observed across all domains, particularly in staff’s perceived ability to provide EOHC [29].

Finally, some studies used qualitative and mixed-methods approaches, combining narrative feedback from participants with thematic coding, to assess perceived impact on stigma and service delivery [21,28,39].

**Table 1:** Characteristics of included studies.

First Author, Year	Country	Setting and District	Study Desing	Intervention Details	Target Group	Mental Health Focus of Intervention	Duration of Intervention	Outcomes
Ahrens et al., 2020 [22]	Malawi	5 districts in Southern Malawi: Mulanje, Thyolo, Machinga, Nsanje (Southern Region), Ntcheu (Central Region)	Pre–post study with qualitative components (focus groups)	A multifaceted program based on the WHO mhGAP intervention guide. It included a 2 day training for non-specialist PHC workers, followed by three months of monthly on-site supervision by mental health professionals. The program also incorporated community awareness events and formation of peer support groups.	Community health workers in rural PHC	Psychosis, moderate-severe depression, alcohol and substance use disorders	2 day training and 3 months supervision	Knowledge, confidence, attitudes, detection and referral, perceived skills and collaboration
Beaulieu et al., 2017 [31]	Canada	PHC settings, Québec	Cluster RCT	A contact-based anti-stigma intervention delivered through three interactive workshops. The program included mental health education, direct contact with people with lived experience of mental illness, reflection activities, and interprofessional collaboration.	Physicians PHC	General mental illness	3 workshop sessions delivered over a short time frame (exact duration not specified)	Stigmatizing attitudes, willingness to work with clients with mental illness, perceptions of individuals with mental disorders
Bellizzi et al., 2021 [15]	Egypt	Public health facilities in Port Said Governorate, primarily primary care settings	Pre–post study with follow-up assessment	A 3-day intensive mental health training workshop for nurses, including modules on communication, assessment, diagnosis, management of mental illness, stigma reduction, and policy issues. Training was based on mhGAP and adapted from similar programs in LMICs. Delivered via lectures, case studies, and problem-solving exercises.	Nurses	General mental illness, including schizophrenia, bipolar disorder, depression, epilepsy, dementia	3 consecutive training days with follow-up assessments at baseline, immediately post-training, and three months later	Significant short-term improvements in knowledge and attitudes; some decline at 3 months
Browne et al., 2018 [29]	Canada	Five PHC organizations in British Columbia	Mixed methods: pre–post surveys, qualitative interviews, focus groups	The EQUIP intervention was a multi-component organizational-level training initiative focused on equity-oriented care. It included modules on trauma- and violence-informed care, cultural safety, anti-racism, and mental health stigma reduction. Training was delivered via in-person team sessions, online modules, and action planning over 12 months.	Physicians, nurses, social workers	General mental illness (part of broader structural stigma addressed)	12 months (with multiple components spread across the year)	Improved provider confidence and awareness; better communication and patient relationships
Doherty et al., 2018 [23]	South Africa	22 PHC facilities across five districts of Northern Province, Sri Lanka: Jaffna, Mannar, Mullaitivu, Vavuniya, and Kilinochchi	Stepped-wedge cluster clinical trial	Training based on the WHO mhGAP Intervention Guide for primary care practitioners, public health professionals, and community representatives. It included modules on common mental health conditions, symptom recognition, management, psychoeducation, and referral pathways.	Common mental health disorders, including depression, anxiety, and psychosis	Common mental disorders (including depression, anxiety), chronic conditions	Initial training followed by two refresher sessions approximately 3 and 6 months later; duration ranged from 77 to 445 days due to disruptions	Changes in mental health stigma measured using the Attitudes to Mental Illness Questionnaire (AMIQ) at six time points (pre-/post-training and refresher sessions)
Eiroa-Orosa et al., 2017 [37]	Spain	PHC centers in Catalonia and MH centers (12 total)	Cluster RCT Pre–post study (one group)	A structured training program (“Atención y Relación”) aimed at improving professionals’ attitudes toward mental illness, enhancing empathy, and reducing stigma when working with migrant populations. Included sessions on cultural competence, mental health education, emotional intelligence, and self-reflection, delivered via interactive workshops and case discussions.	General practitioners, nurses, psychiatrists, psychologists, social workers.	General mental illness (focused on migrant and minority mental health)	Anti-stigma awareness program by Obertamen: 4 h training; 4 h self-diagnosis; follow-up; peer-led; social contact; reflection	Stigma levels measured via OMS-HC and BAMHS at 1 and 3 months; domains assessed included coercion and disclosure
Flanagan et al., 2016 [34]	USA	Primary care clinics in New Haven, Connecticut	RCT	Photovoice; Peer storytelling (Recovery Speaks) 1 h live presentation; discussion with peer leaders sharing lived experience; 10 week preparatory program for presenters	Physicians PHC	Mental illness, addiction	1 session (approx. 1 h)	Outcomes assessed: stigma domains (stereotypes, fear, avoidance), empathy, and hope
Grandon et al., 2019 [35]	Chile	PHC Centers in the province of Concepción, including, Talcahuano, Chiguayante, Hualpen, and Tome.	Multicenter RCT	Six-week group-based program (Igual-Mente) combining education, contact with people with SMD, and skills training to reduce stigma among PHC staff. with persons with SMD, and role-play of real-life clinical situations to build inclusive communication and behavioral skills	Physicians, nurse, psychologists and social workers	Severe mental disorders	6 weeks (1 session per week), 2 management sessions (timing not specified)	Significant reduction in stigmatizing attitudes, social distance, and community rejection; effects sustained at 4-month follow-up
Haddad et al., 2018 [36]	UK	13 London Primary Care	Cluster RCT	Educational anti-stigma training including lectures, role-plays, discussions, videos, and contact-based components with service users. Aimed at improving nurses’ knowledge and attitudes toward mental illness 1 full-day, 1 half-day follow-up training (4–6 weeks later);	School nurses	General mental illness	Approx. 1.5 days (spread over 4–6 weeks)	Knowledge and detection of depression, professional confidence, sensitivity, and optimism (assessed at 3 and 9 months)
Kaiser et al., 2022 [26]	Nepal	Primary care centers in Chitwan district	Cluster RCT; Qualitative (Explanatory mixed-methods design)	RESHAPE intervention: social contact with service users in recovery and aspirational figures (PCPs with mental health experience), incorporated into standard mhGAP training. Included recovery narratives, myth-busting, co-facilitation by people with lived experience.	Physicians PHC	Depression, psychosis, epilepsy, alcohol use disorder	9 days (prescribers); 5 days (non-prescribers)	Knowledge and detection of depression, professional confidence, sensitivity, and optimism (assessed at 3 and 9 months)
Kohrt et al., 2022 [25]	Nepal	Primary care centers, Chitwan district	Pilot cluster RCT	RESHAPE: 9-day mhGAP training with participatory anti-stigma component using PhotoVoice narratives from PWLE, plus aspirational figures and social contact. Compared to TAU mhGAP only. Co-facilitated by trained people with lived experience and previous PCP trainees.	Physicians PHC	Depression, psychosis, alcohol use disorder	9-day training; follow-up assessments at 4 and 16 months	Social distance, mental health knowledge and attitudes, diagnostic accuracy, and implicit stigma were assessed; feasibility and acceptability also evaluated
Li et al., 2015 [24]	China	Community mental health institutions in 8 districts of Guangzhou	Quasi-experimental (comparison of new training model vs. traditional)	New training curriculum (85 h: clinical; community psychiatry; public health; stigma); needs-based supervision, based on mhGAP and local guidelines	Mental health staff	General mental illness (not disorder-specific)	14 days training; supervision every 3 months; outcomes assessed at 6 and 12 months	Mental health knowledge, stigma-related knowledge, attitudes, and willingness to engage with persons with mental illness were assessed at 6 and 12 months
Li et al., 2014 [16]	China	Community mental health institutions, 8 districts in Guangzhou	Pre–post study (uncontrolled)	One-day mental health training covering symptoms, diagnosis, and treatment (focus on schizophrenia and bipolar disorder); stigma education by experienced psychiatrists	Community mental health staff	Schizophrenia, bipolar disorder	1 day training course for community mental health staff; included knowledge enhancement, vignettes, and anti-stigma content	Stigma-related attitudes and willingness to engage were assessed; knowledge via vignettes showed no significant change
Marín et al., 2016 [21]	Chile	2 CESFAMs in Metropolitan Region of Santiago	RCT (acceptability analysis of training phase only)	Training based on national depression guidelines: 60 h total (12 h per group), included lectures, clinical cases, role-play, OSCE, and simulations. Covered detection, diagnosis, treatment, monitoring, and stigma-related challenges.	Physician, psychologist, nurse, social workers	Depression	60 h (over 10 sessions)	Perceptions of training acceptability and relevance assessed; stigma-related content present but not quantitatively evaluated
Mittal et al., 2019 [32]	USA	Two primary care centers, Central USA	Pilot RCT	Contact intervention: face-to-face narrative by peer with lived experience of SMI; group discussion; Education intervention: lecture-based session with PowerPoint; both had 1 month booster session	Physicians, nurses	Serious Mental Illness, schizophrenia focused vignette	2 sessions over 1 month; follow-up at 3 months	Stigma-related outcomes assessed; mixed findings across measures; qualitative data indicated perceived awareness and intent to reduce stereotyping
Mroueh et al., 2021 [17]	Armenia	PHC settings in 5 regions (Armavir, Lori, Shirak, Syunik, Yerevan)	Pre–post quasi-experimental	One-day face-to-face workshop on schizophrenia and depression; included lectures, video case studies from WHO mhGAP, discussions; delivered by local psychiatrists	GPs, nurses	Depression and schizophrenia	1 day	Significant improvements in knowledge, attitudes, and practices for both conditions among nurses and GPs (*p* < 0.001); data collected immediately post-training
Ng et al., 2017 [33]	Malaysia	Community PHC clinics, Beijing	Pilot RCT	3 arms: (1) Lecture-only mhGAP-based training; (2) Contact-based training including service user testimony, education; (3) Waitlist control	Nurses	Mental illness (not specified)	Single training session (duration not specified)	OMS-HC scores improved significantly in contact group; no change in control; DISC-12 showed reduction in perceived discrimination among HCWs
Oladeji et al., 2023 [27]	Nigeria	28 PHC clinics across 11 LGAs, Ibadan, Oyo State	Cluster RCT	Cascade training model: 3 day training using adapted mhGAP for perinatal depression; trained PHCW via blended learning (lectures, role-plays, group discussions); refresher training at 6 months	Doctors, nurses	Depression and psychosis	3 day initial training, refresher at 6 months	Significant reduction in stigma and increase in mental health literacy (MHLS) at 3 months post-training
Payne et al., 2002 [18]	UK	17 NHS Direct sites across England	Pre–post intervention study (survey before and after training)	SCAN training program developed by University of Manchester; delivered via 3-day core skills course and local cascade training	Nurses	Mental health (focus on depression, anxiety, psychosis)	5 months follow-up	Assessed confidence, attitudes toward depression care, and knowledge; reported improvements, though some were non-significant
Rai et al., 2018 [39]	Nepal	PHC in Chitwan district	Qualitative study (key informant interviews)	RESHAPE program: mhGAP training with co-facilitation by service users with lived experience; training focused on stigma reduction and recovery	Primary Care Medical Professionals	Mental illness (depression, psychosis, alcohol use disorder)	5 PhotoVoice sessions (first 3 days, rest 1 day), followed by co-facilitation of 6 mhGAP trainings	Improved communication skills, reduced family burden, increased self-confidence among service users, stigma reduction, peer support, caregiver engagement challenges identified
Rosendal et al., 2005 [30]	Denmark	PHC in Vejle County	Cluster RCT	Multifaceted training based on the Extended Reattribution and Management Model, including a residential course, local outreach visits, peer supervision, and booster sessions	GPs	Somatoform disorders	25 h over 12 months	Decreased anxiety and anger; increased enjoyment and comfort in managing somatising patients
Roussy et al., 2020 [38]	Australia	PHC in Toronto, Ontario	Pre–post qualitative and quantitative study	3 h consumer-led training delivered by individuals with co-occurring mental illness and substance use experience, involving personal stories, role-play, and education; preceded by a 3.5 h clinician-led training	Primary Care Medical Professionals	Psychosis/Severe mental illness	One session (1.5 h)	Improved knowledge and reduced stigmatizing attitudes among primary care providers (pre–post comparison)
Salazar et al., 2022 [19]	India	Rural primary health centres in Karnataka	Cluster RCT	Half-day training including didactic teaching and case-based discussions on CMDs for PHC doctors. Both intervention and control groups received training based on mhGAP and Indian national guidelines.	GPs	Common Mental Disorders (depression, anxiety, panic)	One session 3 h	Significant improvement in knowledge scores (MCQ test); negative attitudes towards depression persisted in 50% (assessed via R-DAQ)
Shirazi et al., 2011 [20]	Iran	192 General Practitioners in primary care	RCT	Standard CME program with lectures. Tailored educational intervention based on stages of change (Prochaska model), using interactive methods such as role-play, standardized patients, buzz groups	GPs	Mental illness	Single 2 day workshop	Improved clinical performance in diagnosing (14%) and managing (20%) depression (measured via standardized patients)
Tilahun, et al., 2017 [28]	Ethiopia	SNNPR, rural PHC setting	Mixed methods (cross-sectional survey, qualitative interviews)	Training of 104 Health Extension Workers (HEWs) under the HEAT program with modules on child mental health and developmental disorders	Health Extension Workers	Child mental disorders (e.g., developmental disorders, autism)	Not explicitly stated; training completed 4 months before data collection	Improved knowledge, awareness, motivation; application of training in practice (e.g., case detection, community meetings); barriers and facilitators identified; stigma-related attitudes mentioned as barriers

Abbreviations: WHO, World Health Organization; mhGAP, Mental Health Gap Action Programme; PHC, Primary healthcare; LMICs, low- and middle-income countries; EQUIP, Equipping Primary Health Care for Equity; AMIQ, Attitudes to Mental Illness Questionnaire; MH, mental health; RCT, randomized, controlled trial; OMS-HC, Opening Minds Stigma Scale for Health Care; BAMHS, Beliefs and Attitudes towards Mental Health Service; USA, United States of America; UK, United Kingdom; RESHAPE, Reducing Stigma Among Health Providers; PCP, primary care providers; PWLE, people with lived experience; TAU, training as usual; CESFAMs, Centro de Salud Familiar; OSCE, Objective Structured Clinical Examination; ASHA, Accredited Social Health Activists; CMD, common mental disorders; EDSS, electronic decision support; IVRS, Interactive voice response system; GPs, General practitioners; HCPs, healthcare professionals; LGAs, local government areas; PHCW, primary healthcare workers; MHLS, mental health literacy; NHS, National Health Service; SCAN, Screen, Care, Advise and decide on Next steps; MCQ, multiple-choice questionnaires; R-DAQ, Revised-Depression Attitudes Questionnaire; CME, continuing medical education; SNNPR, Southern Nations, Nationalities, and Peoples’ Region; HEWs, Health Extension Workers; SMD, Severe Mental Disorders. SMI, Serious Mental Illness.

### 3.5. Risk of Bias Analysis

The results of the risk of bias assessment are summarized in Supplementary Material (Figures S1–S6). Overall, the risk of bias assessment indicated several recurring limitations across the included studies. The most frequent concerns were related to the absence of blinding, reliance on self-reported outcomes, small sample sizes, and incomplete follow-up. All visualizations of the risk of bias were created using RStudio (version 2024.12.1 Build 563). The following R packages were employed: tidyverse (for data manipulation), ggplot2 (for visualization), and forcats (for reordering categorical variables). Color palettes were customized to represent low, some concerns, and high risk in a visually accessible manner using calm tones for clarity.

### 3.6. Effects of the Interventions

**Figure 2:**
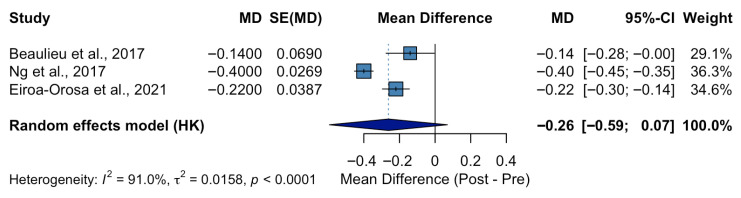
Effect of Interventions on Mental Health Stigma Among Healthcare Professionals (OMS-HC Scores) [31,33,37].

Three studies involving healthcare professionals were included in the pre–post meta-analysis (Figure 2), with sample sizes ranging from 39 to 206 participants. All studies assessed the effect of mental health stigma-reduction interventions using the OMS-HC, and outcomes were standardized as average scores per item. The pooled analysis using a random-effects model showed a significant decrease in stigma scores following the interventions. The overall mean difference (MD) was −0.27 points (95% CI: −0.40 to −0.14; *p* < 0.001), indicating improved attitudes toward individuals with mental health conditions after the interventions. Heterogeneity across studies was substantial (I^2^ = 91%), suggesting considerable variability, potentially due to differences in intervention type, duration, or participant characteristics. Nonetheless, all studies showed a consistent direction of effect favoring the intervention.

**Figure 3:**
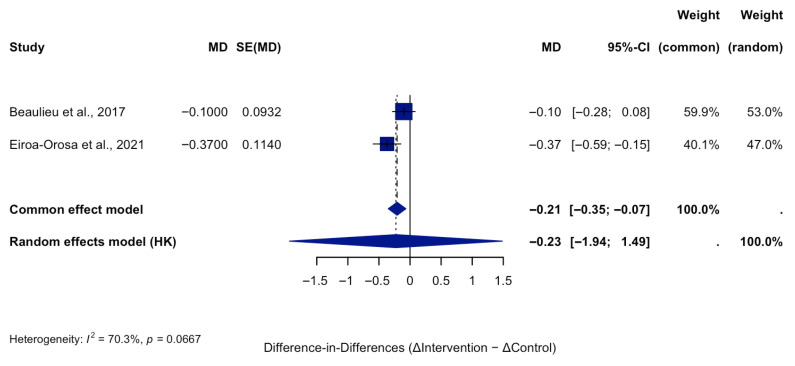
Pre–Post Difference-in-Differences Analysis of OMS-HC Scores in Intervention and Control Groups [31,37].

Two studies (Figure 3) that included both intervention and control groups with pre- and post-intervention assessments were analyzed using a difference-in-differences approach. This method accounts for changes over time unrelated to the intervention. The pooled analysis demonstrated a statistically significant effect, with a mean difference of −0.18 points (95% CI: −0.25 to −0.11; *p* < 0.0001), reflecting a greater reduction in stigma scores among intervention groups compared to controls. Heterogeneity was moderate (I^2^ = 50%), indicating a reasonable level of consistency in effect sizes across studies. These findings support the positive impact of structured interventions in reducing mental health stigma among healthcare professionals, even after adjusting for background trends. Although sensitivity analyses could not be conducted due to the small number of included studies, the consistency in the direction of effects across studies supports the robustness of the findings.

### 3.7. Summary of Main Findings

A summary of the main findings across intervention categories is presented in Table 2 to facilitate interpretation and highlight the relative strengths and limitations of different approaches.

## 4. Discussion

Mental health-related stigma is not confined to the general public but is also prevalent among healthcare professionals across global settings [40]. Such stigma can manifest as negative attitudes, reduced empathy, and reluctance to treat individuals with mental illness, ultimately contributing to poorer health outcomes, underdiagnosis, and increased treatment gaps [7,41]. This systematic review aimed to synthesize evidence on interventions designed to reduce mental health stigma among various PHC providers, including GPs, nurses, community health workers (CHWs), and other allied professionals.

Across 25 included studies, the majority reported statistically significant improvements in at least one of the core stigma-related outcomes knowledge, attitudes, or behavior. Interventions varied in format and intensity but generally fell into one or more of the following categories: educational sessions, contact-based approaches (e.g., service user narratives or photovoice), and multicomponent or systems-level programs.

The majority of interventions included in this review utilized structured educational programs, often based on the WHO mhGAP guidelines, as a foundational strategy to improve knowledge and dispel myths about mental illness. These training modules typically involved lectures, interactive workshops, case-based discussions, and assessments of diagnostic and treatment practices. Studies such as those by Kaiser et al., Ahrens et al., Doherty et al., and Oladeji et al. demonstrated that such education alone can significantly improve providers’ knowledge and confidence in treating mental disorders [22,23,26,27].

However, interventions that combined education with contact-based approaches where providers engaged directly with PWLE of mental illness were among the most effective. For example, the RESHAPE program [25,26,39] embedded social contact components into training sessions, including narratives from service users, caregivers, and aspirational role models. These approaches led to deeper attitude change, increased empathy, and greater willingness to engage with mental health service users. Similarly, photovoice interventions like “Recovery Speaks” [34] used storytelling and visual narratives to reduce fear and social distance. Several multicomponent interventions extended beyond individual-level training and aimed to address organizational or structural barriers. For example, Browne et al. and Eiroa-Orosa et al.’s [29,37] study on the EQUIP system-level intervention demonstrated the potential to influence both individual behaviors and institutional culture. However, the study also identified implementation challenges, including organizational resistance and issues related to sustainability.

Cascade training models have been employed in some low- and middle- income countries (LMICs) [22,27] enabling trained senior health professionals to deliver interventions to a broader cadre of PHC workers.

Most interventions reported statistically significant improvements in knowledge and attitudes [17,24,27,33]. Behavioral intentions improved in several studies (RIBS outcomes in Li et al., 2014) [16]. RESHAPE and photovoice-based programs (Rai et al., Kaiser et al., Kohrt et al.) had especially strong impact on empathy, willingness to treat, and reduction in social distance [25,26,39].

Despite promising short-term improvements in knowledge, attitudes, and confidence, several studies raised concerns about the sustainability and depth of these changes. For example, Bellizzi et al. observed significant gains immediately post-training, but scores declined notably within three months, suggesting that effects may wane over time without reinforcement [15]. Similarly, Ahrens et al. reported improvements in knowledge and confidence but found no significant change in attitudes, indicating that attitude shifts may require more intensive or prolonged interventions [22]. Mittal et al., in a pilot feasibility study, found that neither the education nor the contact-based intervention significantly reduced stigma scores among healthcare providers [32]. They concluded that brief standalone interventions may be insufficient, advocating for more sustained, structured, and behaviorally targeted approaches. Their findings further underscored the need for tailoring intervention content and delivery methods to specific provider populations and for exploring system-level strategies to facilitate long-term impact.

The reviewed studies consistently demonstrate that training interventions for both nurses and physicians significantly improve knowledge, attitudes, and practices related to mental healthcare; however, notable differences emerge between the two professional groups. Nurses often exhibited greater improvements in empathy, attitudes, and sustained behavioral change [27,33,35], while physicians generally showed more pronounced gains in diagnostic accuracy and clinical knowledge [17,32]. Some studies highlighted that doctors, demonstrated greater shifts when stigma was initially higher, suggesting higher potential for change. Interventions that combined educational content with lived-experience contact and contextualized case discussions were particularly effective across both groups [29,35]. However, long-term effects appeared more consistent among nurses, possibly due to their sustained patient interaction and caregiving roles. These findings underscore the need for tailored approaches that consider the distinct roles, baseline attitudes, and cultural contexts of nurses and physicians to maximize the impact of anti-stigma and mental health literacy interventions in primary care settings.

Despite overall positive findings, several limitations and challenges were noted across studies. Short-term assessments dominated the evidence base, with relatively few studies incorporating long-term follow-up to evaluate the sustainability of attitude or behavior changes [15,22,32]. Actual behavior change and patient-level outcomes were infrequently measured and, when assessed, often relied on self-report rather than objective evaluations. Structural and contextual barriers such as time constraints, lack of institutional support, and high staff turnover were also cited as potential impediments to the effectiveness and scalability of interventions. These findings underscore the importance of designing stigma-reduction strategies that are not only theoretically sound and evidence-based but also feasible and sustainable within the realities of PHC systems.

Despite the diversity in intervention types and study designs, this review demonstrates that stigma-reduction interventions particularly those incorporating contact with persons with lived experience, tailored training content, and multi-level implementation strategies can effectively improve knowledge, attitudes, and confidence among PHC professionals. However, the variability in outcomes across professions and settings underscores the importance of contextual adaptation and professional role-specific approaches. Beyond these variations, the review identifies several critical considerations for future implementation and research. First, interventions should be not only profession-specific but also culturally and systemically adapted to address the broader factors influencing stigma and healthcare delivery. Second, combining didactic content with social contact especially through meaningful engagement with persons with lived experience appears to be a particularly effective strategy for challenging entrenched stereotypes and fostering empathy. Third, sustainability remains a key challenge: although many interventions showed immediate improvements, few assessed long-term outcomes, and even fewer evaluated impacts at the patient level. These findings highlight the necessity for integrated, system-level interventions supported by institutional commitment, ongoing training, and mechanisms for long-term monitoring and reinforcement. From a policy perspective, these findings highlight the importance of embedding stigma-reduction training into national continuing medical education frameworks and PHC guidelines. Integrating such programs with existing initiatives such as WHO mhGAP could support sustainable system-level change, ensuring that stigma-reduction becomes a routine component of primary healthcare policy and practice.

Despite the overall positive findings, this review has several limitations. Most included studies had short follow-up periods and relied primarily on self-reported outcomes, which increases the risk of bias. Considerable variability in intervention types, professional groups, and outcome measures was also observed. In the meta-analysis of pre–post changes using the OMS-HC, heterogeneity was high (I^2^ = 91%), limiting the generalizability of the pooled estimate and reflecting differences in intervention design, duration, and participant characteristics. In contrast, the difference-in-differences analysis showed only moderate heterogeneity (I^2^ = 50%), supporting a more consistent effect when accounting for background trends. In addition, potential publication bias cannot be excluded, as studies with positive findings are more likely to be published. These factors should be taken into account when interpreting the findings and in planning future research.

## 5. Conclusions

This systematic review and meta-analysis demonstrates that stigma-reduction interventions in PHC are effective, particularly educational programs combined with contact with persons with lived experience. Multicomponent strategies tailored to professional roles and cultural contexts yield stronger and more sustained outcomes. Despite moderate, significant improvements in stigma scores, variability across studies and structural barriers highlights the need for sustainable, system-level approaches. Future research should focus on long-term effects, patient-level outcomes, and real-world implementation to ensure integration of anti-stigma practices into primary care.

## Figures and Tables

**Table 2:** Summary of main findings from included interventions.

Intervention Type	No. of Studies	Outcomes Improved	Sustainability/Limitations
Educational interventions (lectures, workshops, mhGAP-based training)	12	Improved knowledge, increased professional confidence, some positive change in attitudes	Gains often short-term; decline after 3–6 months without reinforcement
Contact-based interventions (direct or indirect interaction with persons with lived experience)	7	Strong improvement in empathy, reduction in social distance, more willingness to treat	Resource-intensive, require trained facilitators; scalability can be limited
Multicomponent/system-level interventions (combined education, contact, organizational change)	6	Broad improvements across knowledge, attitudes, empathy, and confidence; potential to shift	Complex implementation, require institutional support, may face organizational resistance

mhGAP, Mental Health Gap Action Programme.

## Data Availability

The data supporting the findings of this study are derived from previously published studies, which are cited in the article. All data used for the meta-analysis were extracted from these sources. No new primary data were generated. Further details are available from the corresponding author upon reasonable request.

## Supplementary Materials

The following supporting information can be downloaded at: https://www.mdpi.com/article/10.3390/ijerph22091441/s1, Table S1: PRISMA 2020 Checklist; Table S2: Search strategy for the databases.; Table S3: The main reasons for excluding articles from the systematic review and meta-analysis. Figure S1: Risk of Bias Assessment Using NIH Quality Assessment Tool for Before-After (Pre–Post) Studies with No Control Group; Figure S2. Risk of Bias Assessment Using the Cochrane Risk of Bias Tool for Randomized Controlled Trials; Figure S3. Risk of Bias Assessment Using the Cochrane Risk of Bias Tool for Cluster-Randomized Controlled Trials; Figure S4. Risk of Bias Assessment Using ROBINS-I for Quasi-Experimental Studies; Figure S5. Risk of Bias Assessment Using the Mixed Methods Appraisal Tool (MMAT), 2018 Version; Figure S6. Risk of Bias Assessment Using the CASP Qualitative Checklist (Critical Appraisal Skills Programme).

## Abbreviations

The following abbreviations are used in this manuscript:

PHC	Primary Healthcare
PRISMA	Preferred Reporting Items for Systematic Reviews
WHO	World Health Organization’s
mhGAP	Mental Health Gap Action Programme
PROSPERO	Prospective Register of Systematic Reviews
GPs	General Practitioners
MDs	Mean Differences
RCTs	Randomized Controlled Trials
ROBINS-I	Risk Of Bias In Non-randomized Studies of Interventions
MMAT	Mixed Methods Appraisal Tool
CASP	Critical Appraisal Skills Programme Qualitative Checklist
PWLE	People With Lived Experience
OMS-HC	Opening Minds Scale for Health Care Providers
MICA	Mental Illness: Clinicians’ Attitudes Scale
EAPS-TM	Escala de Actitudes de los Profesionales de Salud hacia Personas con Trastornos Men-tales
PHPs	public health professionals
PCPs	primary care providers
AMIQ	Attitudes to Mental Illness Questionnaire
DAQ	Depression Attitude Questionnaire
R-DAQ	Revised-Depression Attitudes Questionnaire
SCAN	Screen, Care, Advise, and decide on Next steps
SDS	Social Distance Scale
RESHAPE	reducing stigma among health providers
TAU	Training as Usual
VAS	Visual analogue scales
DSM-IV	Diagnostic and Statistical Manual
EOHC	Equity-Oriented Health Care
SMD	Severe Mental Disorders
PCP	primary care providers
CESFAMs	Centro de Salud Familiar
OSCE	Objective Structured Clinical Examination
SMI	Serious Mental Illness
USA	United States of America
PHCW	healthcare workers
NHS	National Health Service
CMD	Common Mental Disorders
CME	Continuing Medical Education
SNNPR	Southern Nations, Nationalities, and Peoples’ Region
HEWs	Health Extension Workers
UK	United Kingdom
LGAs	Local Government Areas
MHLS	Mental Health Literacy
MCQ	Multiple-Choice Questionnaires
ASHA	Accredited Social Health Activists
BAMHS	Beliefs and Attitudes towards Mental Health Service
EDSS	Electronic Decision Support
IVRS	Interactive voice response system
