# Supplementary material for: Interventions to Reduce Mental Health Stigma Among Health Care Professionals in Primary Health Care: A Systematic Review and Meta-Analysis
Source: Int J Environ Res Public Health. 2025 Sep 17;22(9):1441. doi: 10.3390/ijerph22091441 (PMC12469428; doi:10.3390/ijerph22091441)
Supplement: Supplementary file 1 [file ijerph-22-01441-s001.zip › ijerph-3805866-supplementary.pdf]

Supplementary Materials

Interventions to reduce mental health stigma among health care professionals in primary health care: a systematic review and meta-analysis

Lazzat Zhamaliyeva[1], Nurgul Ablakimova[2*], Assemgul Batyrova[3*], Galina Veklenko[3], Andrej M. Grjibovski[4,5,6,7], Sandugash Kudaibergenova[8] and Nursultan Seksenbayev[9]

[1] Department of General Practice No. 2, West Kazakhstan Marat Ospanov Medical University, Aktobe 30012, Kazakhstan; lzhamalieva@mail.ru
[2] Department of Pharmacology, Clinical Pharmacology, West Kazakhstan Marat Ospanov Medical University, Aktobe 030012, Kazakhstan; n.ablakimova@zkmu.kz
[3] Department of Propedeutics of Internal Disease, West Kazakhstan Marat Ospanov Medical University, Aktobe 030012, Kazakhstan; askankyzy@mail.ru; gvictor63@mail.ru
[4] Rectorate, Reaviz Universtiy, 198095 Saint Petersburg, Russia; a.grjibovski@yandex.ru
[5] Department of Epidemiology and Modern Vaccination Technologies, I.M. Sechenov First Moscow State Medical University (Sechenov University), 119048 Moscow, Russia
[6] Department of Healthcare Organization and Preventive Medicine, North-Eastern Federal University, Yakutsk 677000, Russia
[7] Department of Health Policy and Management, Al-Farabi Kazakh National University, Almaty 050040, Kazakhstan
[8] Department of General and Applied Psychology, Al-Farabi Kazakh National University, Almaty, Kazakhstan; sandugash.kudaibergenova@kaznu.kz
[9] Department of Psychiatry and Addiction Medicine, Semey Medical University, Semey 071400, Kazakhstan; nursultan.zhaksylykovich@smu.edu.kz
* Correspondence: askankyzy@mail.ru; n.ablakimova@zkmu.kz

*Assemgul Batyrova: askankyzy@mail.ru;

Supplementary Table S1. PRISMA checklist.

Section and Topic	Item #	Checklist item	Location where item is reported, pp
		**TITLE**	
Title	1	Identify the report as a systematic review.	1
		**ABSTRACT**	
Abstract	2	See the PRISMA 2020 for Abstracts checklist.	3
		**INTRODUCTION**	
Rationale	3	Describe the rationale for the review in the context of existing knowledge.	2-3
Objectives	4	Provide an explicit statement of the objective(s) or question(s) the review addresses.	2-3
		**METHODS**	
Eligibility criteria	5	Specify the inclusion and exclusion criteria for the review and how studies were grouped for the syntheses.	3-4
Information sources	6	Specify all databases, registers, websites, organisations, reference lists and other sources searched or consulted to identify studies. Specify the date when each source was last searched or consulted.	3

Section and Topic	Item #	Checklist item	Location where item is reported, pp
Search strategy	7	Present the full search strategies for all databases, registers and websites, including any filters and limits used.	Suppl. 4-5
Selection process	8	Specify the methods used to decide whether a study met the inclusion criteria of the review, including how many reviewers screened each record and each report retrieved, whether they worked independently, and if applicable, details of automation tools used in the process.	3-4
Data collection process	9	Specify the methods used to collect data from reports, including how many reviewers collected data from each report, whether they worked independently, any processes for obtaining or confirming data from study investigators, and if applicable, details of automation tools used in the process.	4
Data items	10a	List and define all outcomes for which data were sought. Specify whether all results that were compatible with each outcome domain in each study were sought (e.g. for all measures, time points, analyses), and if not, the methods used to decide which results to collect.	3-4
	10b	List and define all other variables for which data were sought (e.g. participant and intervention characteristics, funding sources). Describe any assumptions made about any missing or unclear information.	5
Study risk of bias assessment	11	Specify the methods used to assess risk of bias in the included studies, including details of the tool(s) used, how many reviewers assessed each study and whether they worked independently, and if applicable, details of automation tools used in the process.	5
Effect measures	12	Specify for each outcome the effect measure(s) (e.g. risk ratio, mean difference) used in the synthesis or presentation of results.	4
Synthesis methods	13a	Describe the processes used to decide which studies were eligible for each synthesis (e.g. tabulating the study intervention characteristics and comparing against the planned groups for each synthesis (item #5)).	4
	13b	Describe any methods required to prepare the data for presentation or synthesis, such as handling of missing summary statistics, or data conversions.	4
	13c	Describe any methods used to tabulate or visually display results of individual studies and syntheses.	4
	13d	Describe any methods used to synthesize results and provide a rationale for the choice(s). If meta-analysis was performed, describe the model(s), method(s) to identify the presence and extent of statistical heterogeneity, and software package(s) used.	4
	13e	Describe any methods used to explore possible causes of heterogeneity among study results (e.g. subgroup analysis, meta-regression).	4

Section and Topic	Item #	Checklist item	Location where item is reported, pp
	13f	Describe any sensitivity analyses conducted to assess robustness of the synthesized results.	5
Reporting bias assessment	14	Describe any methods used to assess risk of bias due to missing results in a synthesis (arising from reporting biases).	5
Certainty assessment	15	Describe any methods used to assess certainty (or confidence) in the body of evidence for an outcome.	5
**RESULTS**			
Study selection	16a	Describe the results of the search and selection process, from the number of records identified in the search to the number of studies included in the review, ideally using a flow diagram.	6
	16b	Cite studies that might appear to meet the inclusion criteria, but which were excluded, and explain why they were excluded.	Suppl. 6-7
Study characteristics	17	Cite each included study and present its characteristics.	7-16
Risk of bias in studies	18	Present assessments of risk of bias for each included study.	Suppl. 8-11
Results of individual studies	19	For all outcomes, present, for each study: (a) summary statistics for each group (where appropriate) and (b) an effect estimate and its precision (e.g. confidence/credible interval), ideally using structured tables or plots.	7-17
Results of syntheses	20a	For each synthesis, briefly summarise the characteristics and risk of bias among contributing studies.	7-17, Suppl. 8-11
	20b	Present results of all statistical syntheses conducted. If meta-analysis was done, present for each the summary estimate and its precision (e.g. confidence/credible interval) and measures of statistical heterogeneity. If comparing groups, describe the direction of the effect.	16-17
	20c	Present results of all investigations of possible causes of heterogeneity among study results.	16-17
	20d	Present results of all sensitivity analyses conducted to assess the robustness of the synthesized results.	Suppl. 11
Reporting biases	21	Present assessments of risk of bias due to missing results (arising from reporting biases) for each synthesis assessed.	Suppl. 11
Certainty of evidence	22	Present assessments of certainty (or confidence) in the body of evidence for each outcome assessed.	16-17
**DISCUSSION**			
Discussion	23a	Provide a general interpretation of the results in the context of other evidence.	17-20
	23b	Discuss any limitations of the evidence included in the review.	17-20
	23c	Discuss any limitations of the review processes used.	17-20

Section and Topic	Item #	Checklist item	Location where item is reported, pp
	23d	Discuss implications of the results for practice, policy, and future research.	18-20
**OTHER INFORMATION**			
Registration and protocol	24a	Provide registration information for the review, including register name and registration number, or state that the review was not registered.	3
	24b	Indicate where the review protocol can be accessed, or state that a protocol was not prepared.	3
	24c	Describe and explain any amendments to information provided at registration or in the protocol.	3
Support	25	Describe sources of financial or non-financial support for the review, and the role of the funders or sponsors in the review.	20
Competing interests	26	Declare any competing interests of review authors.	End-matter
Availability of data, code and other materials	27	Report which of the following are publicly available and where they can be found: template data collection forms; data extracted from included studies; data used for all analyses; analytic code; any other materials used in the review.	End-matter

Supplementary Table S2. Search strategy for the databases.

Pubmed		
1	"primary care"[Title/Abstract] OR "primary care health"[Title/Abstract] OR "community health services"[Title/Abstract] OR outpatient[Title/Abstract] OR "family medicine"[Title/Abstract] OR "ambulatory care"[Title/Abstract]	374,333
2	"health personnel"[Title/Abstract] OR nurse*[Title/Abstract] OR doctor*[Title/Abstract] OR physician*[Title/Abstract] OR "medical student*"[Title/Abstract] OR "clinical psychologist"[Title/Abstract] OR "mental health professional"[Title/Abstract] OR "social worker*"[Title/Abstract] OR "community health worker*"[Title/Abstract] OR "healthcare worker*"[Title/Abstract]	1,035,302
3	"stigma"[Title/Abstract] OR "social stigma"[Title/Abstract] OR "mental health stigma" "stereotyping"[Title/Abstract] OR "attitude"[Title/Abstract] OR "negative attitudes"[Title/Abstract] OR "discrimination"[Title/Abstract] OR "social distance"[Title/Abstract]	255,269
4	"mental disorder*"[Title/Abstract] OR depression*[Title/Abstract] OR anxiety[Title/Abstract] OR "mental illness"[Title/Abstract] OR "psychiatric disorders"[Title/Abstract]	757,608
5	intervention[Title/Abstract] OR education[Title/Abstract] OR training[Title/Abstract] OR program[Title/Abstract] OR	2,994,077

	teaching[Title/Abstract] OR "contact-based education"[Title/Abstract] OR "video intervention"[Title/Abstract] OR "anti-stigma campaign"[Title/Abstract] OR learning[Title/Abstract] OR "awareness raising"[Title/Abstract]	
6	#1 AND #2 AND #3 AND #4 AND #5	86
7	#1 AND #2 AND #3 AND #4 AND #5 Article (Document Types) and English (Languages)	9
Scopus		
1	TITLE-ABS-KEY ("primary care" OR "primary care health" OR "community health services" OR outpatient OR "family medicine" OR "ambulatory care")	627,129
2	TITLE-ABS-KEY ("health personnel" OR nurse* OR doctor* OR physician* OR "medical student*" OR "clinical psychologist" OR "mental health professional" OR "social worker*" OR "community health worker*" OR "healthcare worker*")	1,957,513
3	TITLE-ABS-KEY ("stigma" OR "social stigma" OR "mental health stigma" "stereotyping" OR "attitude" OR "negative attitudes" OR "discrimination" OR "social distance")	30,736
4	TITLE-ABS-KEY ("mental disorder*" OR depression* OR anxiety OR "mental illness" OR "psychiatric disorders")	1,578,518
5	TITLE-ABS-KEY (intervention OR education OR training OR program OR teaching OR "contact-based education" OR "video intervention" OR "anti-stigma campaign" OR learning OR "awareness raising")	11,153,008
6	#1 AND #2 AND #3 AND #4 AND #5	184
7	#1 AND #2 AND #3 AND #4 AND #5 AND (LIMIT-TO (DOCTYPE, "ar")) AND (LIMIT-TO (LANGUAGE , "English"))	157
Web of science		
1	"primary care" OR "primary health care" OR "primary healthcare" OR "community health services" OR outpatient* OR "family medicine" OR "ambulatory care" (Title) or "primary care" OR "primary health care" OR "primary healthcare" OR "community health services" OR outpatient* OR "family medicine" OR "ambulatory care" (Abstract) or "primary care" OR "primary health care" OR "primary healthcare" OR "community health services" OR outpatient* OR "family medicine" OR "ambulatory care" (Keyword Plus ®)	445,304
2	((TI=("health personnel" OR nurse* OR doctor* OR physician* OR "medical student*" OR "clinical psychologist" OR "mental health professional" OR "social worker*" OR "community health worker*" OR "healthcare worker*")) OR AB=("health personnel" OR nurse* OR doctor* OR physician* OR "medical student*" OR "clinical psychologist" OR "mental health professional" OR "social worker*" OR "community health worker*" OR "healthcare worker*")) OR AK=("health personnel" OR nurse* OR doctor* OR physician* OR "medical student*" OR "clinical psychologist" OR "mental health professional" OR "social worker*" OR "community health worker*" OR "healthcare worker*")	894,126
3	((TI=("stigma" OR "social stigma" OR "mental health stigma" "stereotyping" OR "attitude" OR "negative attitudes" OR	536,263

	"discrimination" OR "social distance")) OR AB=("stigma" OR "social stigma" OR "mental health stigma" "stereotyping" OR "attitude" OR "negative attitudes" OR "discrimination" OR "social distance")) OR AK=("stigma" OR "social stigma" OR "mental health stigma" "stereotyping" OR "attitude" OR "negative attitudes" OR "discrimination" OR "social distance")	
4	((TI=("mental disorder*" OR depression* OR anxiety OR "mental illness" OR "psychiatric disorders")) OR AB=("mental disorder*" OR depression* OR anxiety OR "mental illness" OR "psychiatric disorders")) OR AK=("mental disorder*" OR depression* OR anxiety OR "mental illness" OR "psychiatric disorders")	900,265
5	((TI=(intervention OR education OR training OR program OR teaching OR "contact-based education" OR "video intervention" OR "anti-stigma campaign" OR learning OR "awareness raising")) OR AB=(intervention OR education OR training OR program OR teaching OR "contact-based education" OR "video intervention" OR "anti-stigma campaign" OR learning OR "awareness raising")) OR AK=(intervention OR education OR training OR program OR teaching OR "contact-based education" OR "video intervention" OR "anti-stigma campaign" OR learning OR "awareness raising")	7,791,254
6	#1 AND #2 AND #3 AND #4 AND #5	318
7	#1 AND #2 AND #3 AND #4 AND #5 AND Article (Document Types)	296
8	#1 AND #2 AND #3 AND #4 AND #5 AND Article (Document Types) and English (Languages)	294

Supplementary Table S3. The main reasons for excluding articles from the systematic review and meta-analysis.

№	Study reference	Reason for exclusion
1	R. A. Hana, E. Heim, P. Cuijpers, M. Sijbrandij, R. E. Chammay and B. A. Kohrt. Addressing “what matters most” to reduce mental health stigma in primary healthcare settings: a qualitative study in Lebanon. BMC Prim Care. 2024 Dec 19;25:427.	Not an implemented intervention; exploratory phase. The study is at the conceptualization stage; it describes potential WM-based approaches, but does not test the intervention or evaluate the effect.
2	X. T. He, S. B. Chen, Q. Y. Zhang, S. He, L. Yang and J. Ma. Mental Health Literacy and Professional Psychological Help-Seeking Attitudes Among Primary Healthcare Workers: The Mediating Role of Social Support and Mental Illness Stigma. Risk Manag Healthc Policy. 2025 Feb 28;18:703-718	No implemented intervention cross-sectional observational study without an actual intervention to reduce mental health stigma
3	K. Sanchez, M. O. Killian, B. H. Eghaneyan, L. J. Cabassa and M. H. Trivedi. Culturally adapted depression education and engagement in treatment among Hispanics in primary care: outcomes from a pilot feasibility study. Bmc Family Practice 2019 Vol. 20 Issue 1 Pages 9.	Target population: patients, not healthcare professionals.<br>Intervention delivered to patients, not healthcare providers.
4	K. Searle, G. Blashki, R. Kakuma, H. Yang, Y. Zhao and H. Minas. Current needs for the improved management of depressive disorder in community healthcare centres, shenzhen, china: A view from primary care medical	Population not eligible: participants were healthcare managers or leaders, not frontline providers delivering direct patient care.

	leaders. International Journal of Mental Health Systems 2019 Vol. 13 Issue 1 Pages 1-18.	Intervention not implemented: the study did not evaluate or implement a stigma-reduction intervention, but explored perceptions and barriers.<br>Study design not eligible: purely qualitative needs assessment with no intervention or outcome evaluation.
5	O. Selohilwe, A. Bhana, E. C. Garman and I. Petersen. Evaluating the role of levels of exposure to a task shared depression counselling intervention led by behavioural health counsellors: outcome and process evaluation. International Journal of Mental Health Systems 2019 Vol. 13 Pages 15	Population not eligible intervention targeted patients, not healthcare providers
6	A. Tewari, S. Kallakuri, S. Devarapalli, V. Jha, A. Patel and P. K. Maulik. Process evaluation of the systematic medical appraisal, referral and treatment (SMART) mental health project in rural India. Bmc Psychiatry 2017 Vol. 17 Pages 13.	Population not eligible - participants were women from rural communities, not healthcare professionals.<br>Intervention not targeting health professionals - focused on awareness and empowerment of community members.<br>Setting not in primary healthcare - no intervention directed at PHC providers.<br>Outcomes not eligible - no stigma-related outcomes measured among healthcare staff.
7	V. D. Tomaras, M. Ginieri-Coccossis, M. Vassiliadou, M. Malliori, S. Ferentinos, C. R. Soldatos, et al. Education in mental health promotion and its impact on the participants' attitudes and perceived mental health. Annals of General Psychiatry 2011 Vol. 10 Pages 10.	Population not eligible: The study participants were not frontline healthcare providers in PHC settings but a mixed group (e.g., judges, educators, police, clergy, journalists).<br>Setting not eligible: Intervention was not implemented in primary health care or outpatient clinical settings.<br>Intervention focus not specific to stigma: General mental health promotion education without a clearly defined stigma-reduction focus for healthcare workers.
8	P. K. Maulik, S. Kallakuri, S. Devarapalli, V. K. Vadlamani, V. Jha and A. Patel. Increasing use of mental health services in remote areas using mobile technology: a pre-post evaluation of the SMART Mental Health project in rural India. Journal of Global Health 2017 Vol. 7 Issue 1 Pages 13.	Population data not disaggregated stigma outcomes were reported for a mixed group (including health professionals and non-clinical community leaders), with no separate results available for primary health care professionals.

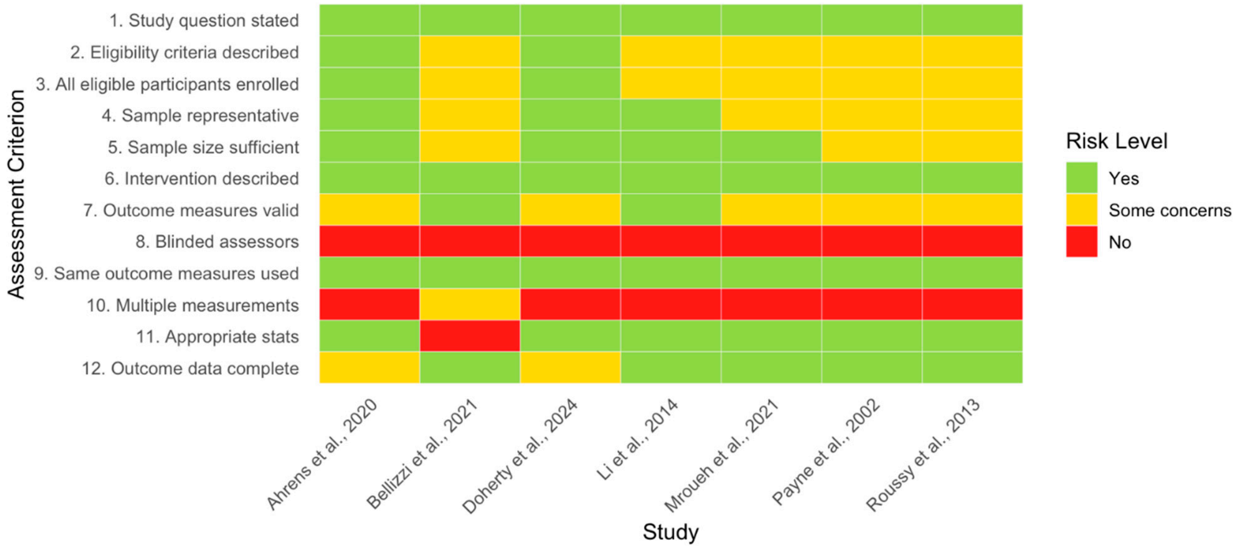

Supplementary Figure S1. Risk of Bias Assessment Using NIH Quality Assessment Tool for Before-After (Pre-Post) Studies With No Control Group

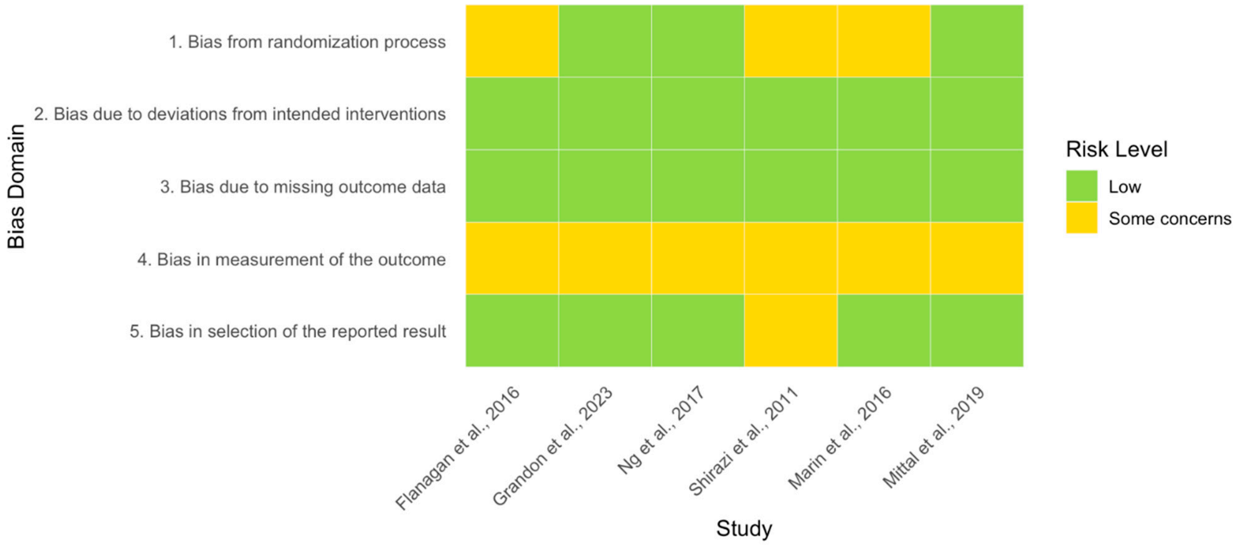

Supplementary Figure S2. Risk of Bias Assessment Using the Cochrane Risk of Bias Tool for Randomized Controlled Trials

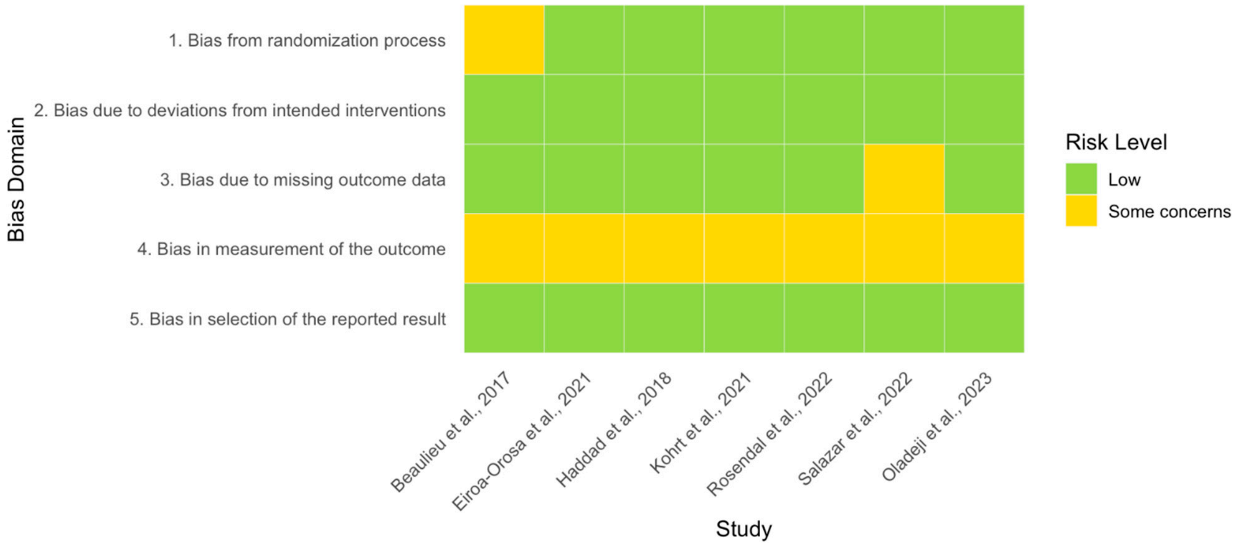

Supplementary Figure S3. Risk of Bias Assessment Using the Cochrane Risk of Bias Tool for Cluster-Randomized Controlled Trials

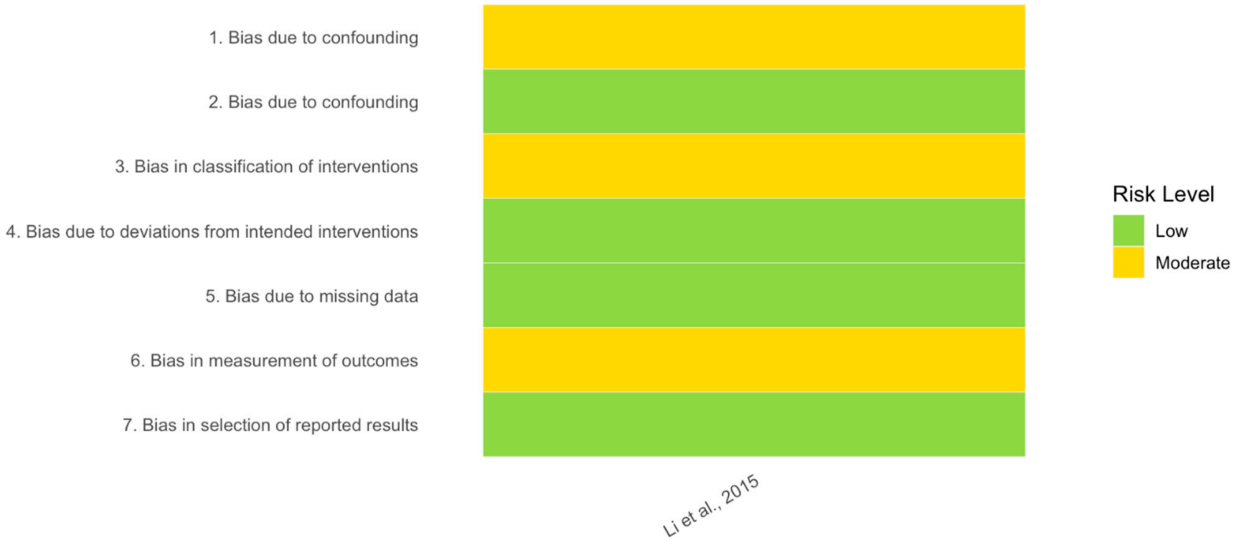

Supplementary Figure S4. Risk of Bias Assessment Using ROBINS-I for Quasi-Experimental Studies

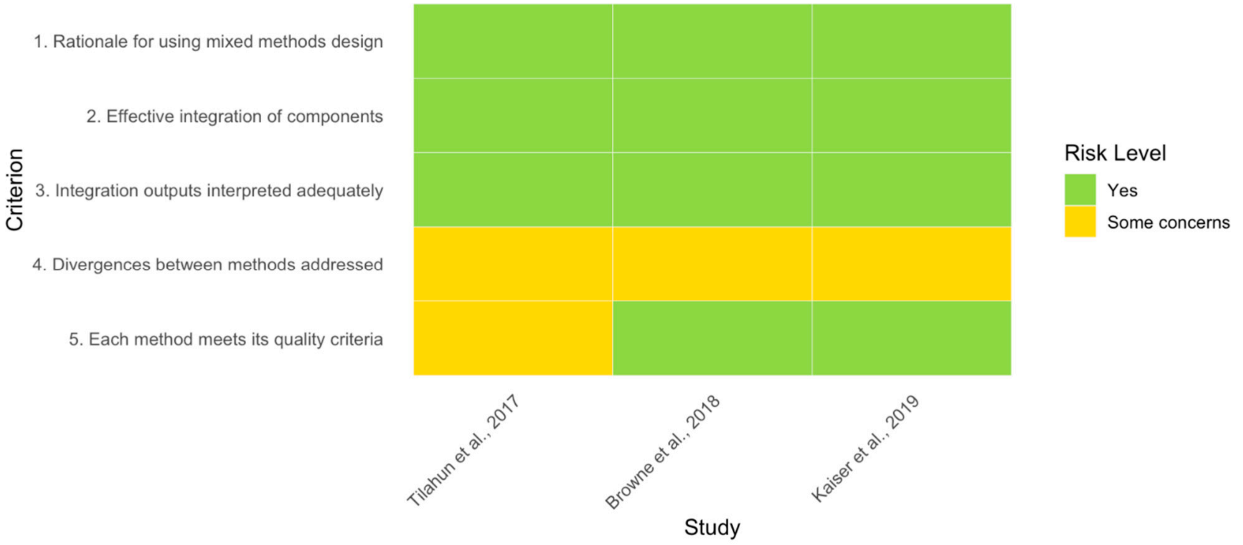

Supplementary Figure S5. Risk of Bias Assessment Using the Mixed Methods Appraisal Tool (MMAT), 2018 Version

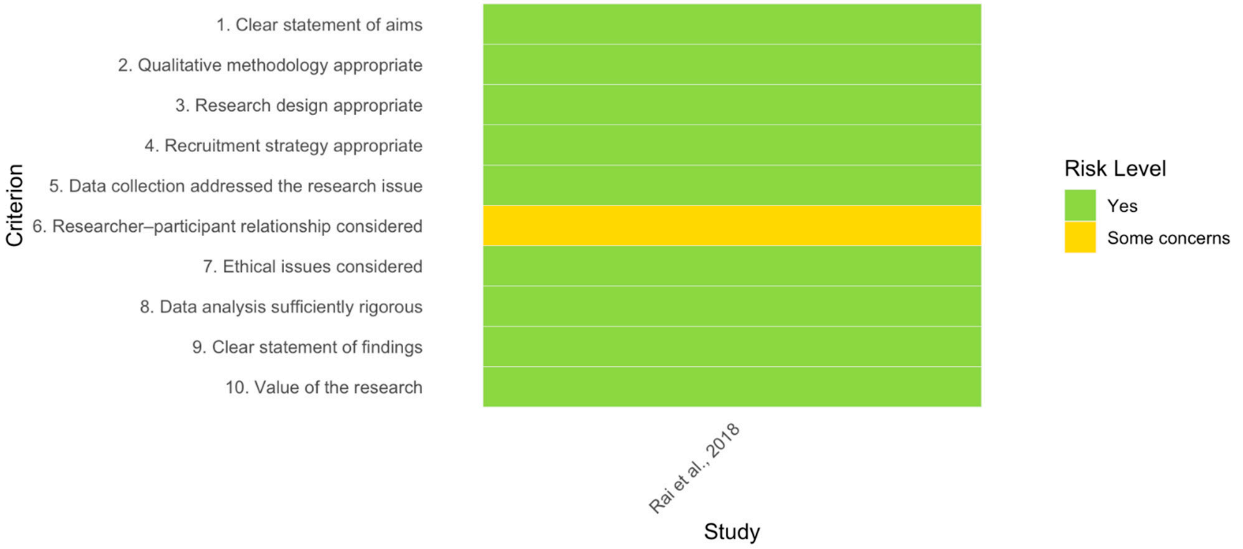

Supplementary Figure S6. Risk of Bias Assessment Using the CASP Qualitative Checklist (Critical Appraisal Skills Programme)

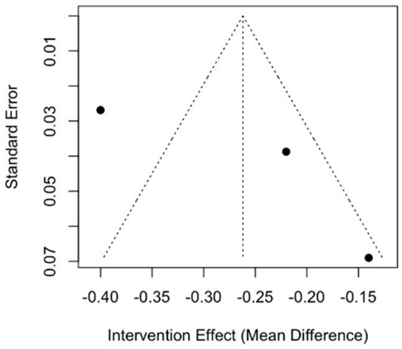

Supplementary Figure S7. Forest plot of pre-post intervention effects on stigma scores (OMS-HC) Figure 3.

Funnel plot assessing publication bias in pre-post intervention studies
Visual inspection shows moderate asymmetry, but Egger's test p = 0.206, indicating no significant small-study effects. Contour lines represent conventional significance levels (90%, 95%, 99%). Symmetry suggests a low risk of publication bias.

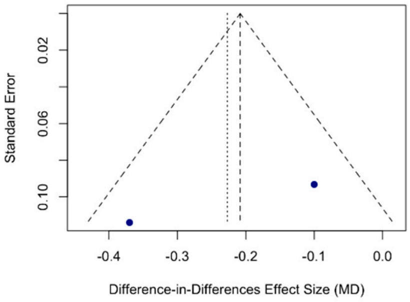

Supplementary Figure S8. Forest plot of difference-in-differences effects in studies with control groups (OMS-HC).

Funnel plot assessing publication bias in DiD studies with control groups
Limited to two studies; interpretability is restricted. Visual symmetry observed, but statistical tests (e.g., Egger's) not applicable with <3 studies.
